# Supplementary material for: Bayesian refinement of protein structures and ensembles against SAXS data using molecular dynamics
Source: PLoS Comput Biol. 2017 Oct 18;13(10):e1005800. doi: 10.1371/journal.pcbi.1005800 (PMC5662244; doi:10.1371/journal.pcbi.1005800)
Supplement: S1 Archive — (BZ2) [file pcbi.1005800.s011.tar.bz2 › gromacs_4.62_bayesian_saxs_refi/share/html/online/g_sigeps.html]

g\_sigeps


| g\_sigeps Main Table of Contents |

**VERSION 4.6  
Sat 19 Jan 2013**

---

### Description

g\_sigeps is a simple utility that converts C6/C12 or C6/Cn combinations
to σ and ε, or vice versa. It can also plot the potential
in file. In addition, it makes an approximation of a Buckingham potential
to a Lennard-Jones potential.

### Files

| option | filename | type | description |
| --- | --- | --- | --- |
| **-o** | potje.xvg | Output | xvgr/xmgr file |

### Other options

| option | type | default | description |
| --- | --- | --- | --- |
| **-[no]h** | bool | no | Print help info and quit || **-[no]version** | bool | no | Print version info and quit || **-nice** | int | 0 | Set the nicelevel || **-[no]w** | bool | no | View output .xvg, .xpm, .eps and .pdb files || **-xvg** | enum | xmgrace | xvg plot formatting: xmgrace, xmgr or none || **-c6** | real | 0.001 | C6 || **-cn** | real | 1e-06 | Constant for repulsion || **-pow** | int | 12 | Power of the repulsion term || **-sig** | real | 0.3 | σ || **-eps** | real | 1 | ε || **-A** | real | 100000 | Buckingham A || **-B** | real | 32 | Buckingham B || **-C** | real | 0.001 | Buckingham C || **-qi** | real | 0 | qi || **-qj** | real | 0 | qj || **-sigfac** | real | 0.7 | Factor in front of σ for starting the plot |

---

http://www.gromacs.org  
gromacs@gromacs.org
